# Genetic architecture and polygenic risk score prediction of degenerative suspensory ligament desmitis (DSLD) in the Peruvian Horse

**DOI:** 10.3389/fgene.2023.1201628

**Published:** 2023-08-14

**Authors:** Mehdi Momen, Kiley Brauer, Margaret M. Patterson, Susannah J. Sample, Emily E. Binversie, Brian W. Davis, E. Gus Cothran, Guilherme J. M. Rosa, Sabrina H. Brounts, Peter Muir

**Affiliations:** ^1^ Department of Surgical Sciences, School of Veterinary Medicine, University of Wisconsin-Madison, Madison, WI, United States; ^2^ Department of Veterinary Integrative Biosciences, College of Veterinary Medicine and Biomedical Sciences, Texas A&M University, College Station, TX, United States; ^3^ Department of Animal and Dairy Sciences, College of Agriculture and Life Sciences, University of Wisconsin-Madison, Madison, WI, United States

**Keywords:** degenerative suspensory ligament desmitis, DSLD, Peruvian Horse, genome-wide association study, GWAS, genetic architecture, polygenic risk score prediction, biological pathways

## Abstract

**Introduction:** Spontaneous rupture of tendons and ligaments is common in several species including humans. In horses, degenerative suspensory ligament desmitis (DSLD) is an important acquired idiopathic disease of a major energy-storing tendon-like structure. DSLD risk is increased in several breeds, including the Peruvian Horse. Affected horses have often been used for breeding before the disease is apparent. Breed predisposition suggests a substantial genetic contribution, but heritability and genetic architecture of DSLD have not been determined.

**Methods:** To identify genomic regions associated with DSLD, we recruited a reference population of 183 Peruvian Horses, phenotyped as DSLD cases or controls, and undertook a genome-wide association study (GWAS), a regional window variance analysis using local genomic partitioning, a signatures of selection (SOS) analysis, and polygenic risk score (PRS) prediction of DSLD risk. We also estimated trait heritability from pedigrees.

**Results:** Heritability was estimated in a population of 1,927 Peruvian horses at 0.22 ± 0.08. After establishing a permutation-based threshold for genome-wide significance, 151 DSLD risk single nucleotide polymorphisms (SNPs) were identified by GWAS. Multiple regions of enriched local heritability were identified across the genome, with strong enrichment signals on chromosomes 1, 2, 6, 10, 13, 16, 18, 22, and the X chromosome. With SOS analysis, there were 66 genes with a selection signature in DSLD cases that was not present in the control group that included the *TGFB3* gene. Pathways enriched in DSLD cases included proteoglycan metabolism, extracellular matrix homeostasis, and signal transduction pathways that included the hedgehog signaling pathway. The best PRS predictive performance was obtained when we fitted 1% of top SNPs using a Bayesian Ridge Regression model which achieved the highest mean of R^2^ on both the probit and logit liability scales, indicating a strong predictive performance.

**Discussion:** We conclude that within-breed GWAS of DSLD in the Peruvian Horse has further confirmed that moderate heritability and a polygenic architecture underlies the trait and identified multiple DSLD SNP associations in novel tendinopathy candidate genes influencing disease risk. Pathways enriched with DSLD risk variants include ones that influence glycosaminoglycan metabolism, extracellular matrix homeostasis, signal transduction pathways.

## 1 Introduction

**FIGURE 1:**
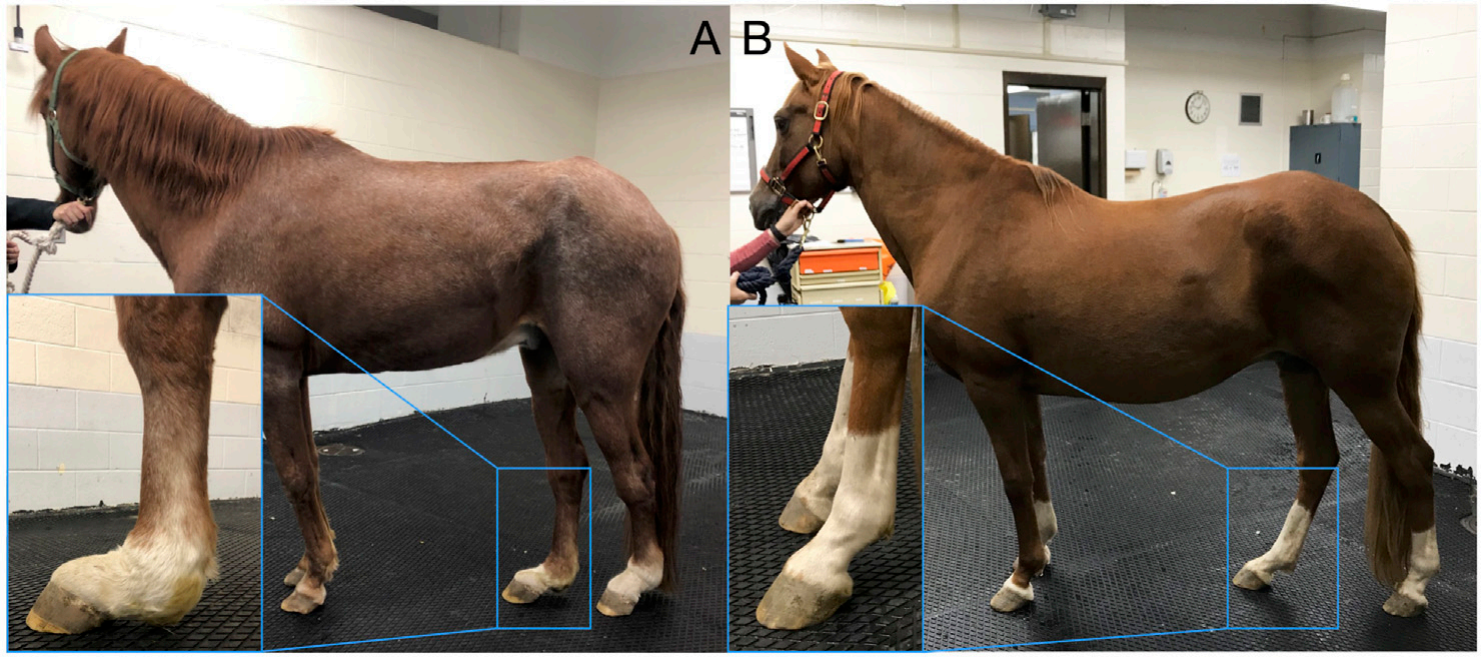
Degenerative suspensory ligament desmitis (DSLD) is a crippling, painful equine disease. **(A)** A Peruvian Horse that is severely affected with DSLD and a **(B)** phenotype-negative control Peruvian Horse. As the disease develops, the suspensory ligament (SL) progressive thickens. Over time, the SL mechanically weakens and ruptures, resulting in a classic sign of dropped fetlocks. In the severe case, obvious thickening and dropping of the fetlocks is evident (inset A) compared with the normal standing posture (inset B). DSLD is typically more evident in the pelvic limbs verses the thoracic limbs, although in some Peruvian Horses DSLD develops in all four limbs. Reproduced from Momen et al., 2022.

Spontaneous rupture of tendons and ligaments in response to trauma or chronic degeneration is a common injury shared across species. In humans, rotator cuff and Achilles’ tendon injuries are common diseases that often lead to chronic tendon degeneration (Thomopoulos et al., 2015). In horses, degenerative suspensory ligament (SL) desmitis (DSLD) is an idiopathic, devastating disease of an essential energy-storing tendon-like structure that prevents hyperextension of the fetlock joint (Mero and Pool, 2002). Typically, a multi-limb disease, horses affected with DSLD experience progressive hyperextension of their fetlocks because of degeneration and rupture of the SL and its distal branches, resulting in lameness and a decreased quality of life (Mero and Scarlett, 2005). Histologically, collagen disruption, accumulation of interfibrillar proteoglycans in ligament matrix, and chondroid metaplasia are key pathological features in affected horses (Halper et al., 2006; Plaas et al., 2011). Age at diagnosis is in the range of ∼5–10 years and often results in euthanasia due to the life-limiting lameness associated with dropped fetlocks (Figure 1). The Peruvian Horse (Peruvian Paso), Paso Fino, Warmblood, Morgan, and Akhal-Teke breeds are predisposed to DSLD, whilst ponies and draft breeds have reduced disease risk (Mero and Scarlett, 2005). In some Peruvian Horse families, the incidence may be as high as 40%, suggesting familial association (Mero and Scarlett, 2005). The late onset of the disease means that DSLD-affected horses have often been used for breeding before clinical signs of tendon/ligament injury (TLI) develop, causing economic loss. Clinically, it is recommended not to use affected horses for breeding.

Presently, there is little understood about the mechanism leading to DSLD. Strong breed disposition suggests a substantial genetic contribution to risk of DSLD. However, the genetic architecture of DSLD is unclear. To date, no estimate of DSLD heritability has been reported in any horse breed. Currently, there is no genetic test available that could assess a horse’s risk of developing DSLD.

In the Peruvian Horse, as in other horse breeds, domestication and breed development has generated selective pressures on the genome to enable horses to work in agriculture, and transport. More recently, traits such as morphology and performance have been considered during selection for breeding (Petersen et al., 2013; Gouveia et al., 2014). These genetic differentiation events have been evolutionarily generated by natural and artificial selection. An unintended consequence of breed development is the increased incidence of disease within individual breeds or breed groups. Many spontaneous diseases in horses closely mimic heritable disorders seen in humans but occur in a model where reduced genetic diversity within a breed can generate long stretches of linkage disequilibrium (LD). In this regard equine DSLD is an important spontaneous model of chronic human TLI that is often related to disturbances in matrix homeostasis.

During past decades, attempts have been made to discover the genetic background of DSLD in the Peruvian Horse. These studies involved investigation of the molecular pathology of DSLD, disturbances to signaling pathways, a genome-wide association study (GWAS) with low density markers, and case-control differential gene expression analysis (Mero and Pool, 2002; Strong, 2005; Young et al., 2018; Haythorn et al., 2020). Whether a simple or polygenic architecture explains the genetic contribution to this important equine disease remains unclear. Earlier work using low-resolution GWAS in the Peruvian Horse identified candidate loci on chromosomes 6, 7, 11, 14, 26 that did not meet genome-wide significance (Strong, 2005; Metzger and Distl, 2020). Improved understanding of the genetic contribution to DSLD is clearly needed. Initial observational studies of breed predisposition suggest that DSLD-associated genetic variants are enriched in the Peruvian Horse through linkage to desirable phenotypes. From an evolutionary point of view, selection for a desired phenotype through careful breeding results in an increased frequency of haplotypes containing the gene(s) and functional allele(s) conferring the phenotype at a rate greater than expected under a null model of neutral evolution (Cutter and Payseur, 2013). GWAS and detection of signatures of selection (SOS) are two common genetic analysis approaches for case control association between a disease phenotype and genetic markers, typically single nucleotide polymorphisms (SNPs) (Patron et al., 2019; Momen et al., 2022).

Consequently, we recruited a reference population of Peruvian Horses phenotyped as DSLD cases or controls, undertook a GWAS and SOS analysis, and used the reference population to undertake polygenic risk score (PRS) prediction of disease risk. Discovery of strong DSLD candidate loci and genes influencing disease risk would represent a significant advance. Furthermore, we estimated heritability using a population-based pedigree to assess narrow-sense heritability. Identification of genomic regions that contribute to the genetic risk of DSLD will permit development of a genetic screening test to assess risk of DSLD in Peruvian Horses. Additionally, gene mutations that influence risk of DSLD in the Peruvian Horse represent important candidate genes for risk of human and canine spontaneous TLI and rupture. We confirmed moderate heritability and a complex genetic architecture for DSLD in the Peruvian Horse and show that PRS prediction using Bayesian ridge regression (BRR) is highly accurate at predicting risk of DSLD in this breed.

## 2 Materials and methods

### 2.1 Recruitment and phenotyping

Client-owned Peruvian Horses were recruited at the UW Madison School of Veterinary Medicine and Texas A&M College of Veterinary Medicine and through online advertising. Hair bulb samples pulled from the tail or mane, nasal swabs, or EDTA blood samples were collected from 183 Peruvian Horses for case control binary GWAS. The data set consisted of 80 cases and 103 controls. All owners gave informed consent to participate in the study. All procedures were performed in accordance with the recommendations in the Guide for the Care and Use of Laboratory Animals of the National Institutes of Health and the American Veterinary Medical Association and with approval from the Animal Care Committee of the University of Wisconsin-Madison (Protocols V1070, V5463) and Texas A&M University (Protocol AUP IACUC 2018-0443 CA). Preparation of the manuscript conformed with the ARRIVE guidelines. If available, a pedigree was collected from each horse to confirm purebred status. DSLD cases were diagnosed with information from the veterinary records, such as physical exam findings, lameness exam findings and photographs. Physical examination consisted of palpation of soft tissue structures in all four distal limbs for pain, swelling, heat, asymmetry or scarring. Range-of-motion and resistance to manipulation was assessed. Fetlock flexion after gait evaluation at the walk and trot on hard and soft surfaces was evaluated. As DSLD develops, the SL progressively thickens. Over time, the SL mechanically weakens and ruptures, resulting in a classic sign of dropped fetlocks in multiple limbs. In the severe case, obvious thickening and dropping of the fetlocks is evident compared with the normal standing posture (Figure 1). In horses with DSLD, the SL progressively weakens causing hyperextension of the fetlock, hock, and stifle. In some cases, ultrasound examination further confirmed the disease-status. B-mode tendon ultrasound examination of the SL using a linear 12 MHz transducer to include both the medial and lateral branches (Mero and Scarlett, 2005) can provide additional confirmation of DSLD. If necessary, sedation with xylazine or detomidine/butorphanol was given to the horse to facilitate the examination. Control horses were normal on physical exam and ≥15 years, as onset of DSLD in horses in this age range is unlikely (Mero and Scarlett, 2005). If a control horse developed DSLD through follow-up contact with the owner, its phenotype was updated. Medical records were also reviewed for the presence of other diseases that could be associated with development of tendon laxity, although this did not lead to inclusion or exclusion of a subject horse.

### 2.2 DNA isolation and SNP genotyping

DNA was isolated from buffy coat, hair bulbs obtained from the mane or tail, or nasal swabs (Performagene PG-100, DNA Genotek, Ottawa, Canada). Blood was collected in EDTA-coated tubes. For genotyping, samples underwent DNA isolation using the Gentra Puregene kit (Qiagen, Valencia, CA, United States). DNA quantity and purity were assessed using a Qubit 4 Fluorometer (Thermo Scientific, Waltham, MA, United States) and a Nanodrop Lite Spectrophotometer (Thermo Scientific, Waltham, MA, United States).

Isolated DNA samples were stored at −20°C until genotyping. SNP genotyping was performed using the Axiom Equine Genotyping Array (Axiom MNEC670K, Thermo Scientific, Waltham, MA, United States) which includes a total of 670,796 SNPs. Genomic coordinates based on the latest version of genome assembly, EquCab3.0 SNP collection (https://www.ncbi.nlm.nih.gov/assembly/GCF_002863925.1/), was used throughout the study.

### 2.3 Imputation missing genotypes and SNP filtering

Missing genotypes were imputed using the Beagle software, version 5.4 (Browning & Browning, 2007). The software uses a hidden Markov model (HMM) to construct a tree of haplotypes and summarize it in a direct acyclic graph by joining nodes of the tree based on haplotype similarity to infer missing markers. Quality control was performed using PLINK v1.9 (Chang et al., 2015). SNPs were removed from the dataset if they had minor allele frequency (MAF) < 0.05, SNP genotyping call rate <95%, individual horse call rate of <90%, or did not conform to Hardy-Weinberg proportions at *P* < 1E-06. After quality control, 177,662 SNPs were removed, and 447,630 SNPs remained for analysis.

### 2.4 Heritability estimation from pedigrees

The Peruvian Horse dataset included a total of 1,947 individuals of which there were 54 horses with a case phenotype and 116 horses with control phenotype. Among these, there were 607 individuals with progeny acting as sires and 963 individual females with progeny and the rest consisting of individuals without any recorded progeny. The dataset consisted of 70 full-sibling groups, with an average family size of 2.19 individuals per group. The CFC software tool (Sargolzaei et al., 2006) was used to analyze the structure of the pedigree. Inbreeding was determined by the pedigree relationship coefficient (F), which was computed from the diagonal elements of the numerator relationship matrix $F_{j}=A_{jj}-1$ , where **A** is the pedigree relationship matrix. The threshold for inbreeding was 1.0. A probit Bayesian linear mixed model, using the MCMCglmm package (Hadfield, 2010), was then used to generate a posterior distribution of heritability for DSLD in the Peruvian Horse. The MCMC chain was run for a total of 1,000,000 iterations plus a burn-in of 20,000 samples and a thinning interval of 5, meaning there were 100,000 posterior probabilities sampled of the variance components.

### 2.5 Genome-wide association study

A univariate logistic linear mixed regression model in R as implemented by the ‘gaston’ package (Perdry and Dandine-Roulland, 2020) was used for the association analysis. Each SNP was regressed using the Wald test and sex was used as a covariate. *P*-values were examined to assess the significance of SNP associations with DSLD. A genomic relationship matrix (GRM) as formulated by VanRaden (2008), was used to account for population stratification and relatedness among individuals:

$$G=\frac{XX^{\prime }}{2\Sigma p_{i}\left(1-p_{i}\right)}$$

Where, where **X** is an *n* by *m* matrix of centered genotypes and $p_{i}$ is the minor allele frequency for allele i.

Two different *P*-value thresholds were considered. One threshold was determined through a Bonferroni correction (*p* < 0.05/total number of SNPs). As an alternative approach, we established genome-wide significance thresholds using 95% confidence intervals (CI) derived from the empirical distribution of *P*-values obtained under the null hypothesis of no association (Karlsson et al., 2013). To construct this distribution, we performed 500 permutations of the phenotypes and reran the GWAS each time. Genome-wide significance was defined as associations surpassing the upper 5% empirical CI, corresponding to a *P*-value threshold of ≤7.39E−05. A Quantile-Quantile plot was made to compare expected null distribution of the test statistic with the observed genome-wide based distribution. A list of GWAS genes was built using the EquCab3.0 genome assembly and the Ensembl genome browser. Regions of the reference genome were scanned ±50 kb upstream and downstream from the positions of SNPs that crossed the Bonferroni significance threshold. Associated genes were then investigated for relevance to tendon homeostasis using PubMed and the search term “tendon”.

### 2.6 Regional window variance using local genomic partitioning

Regional variance analysis enhances the power to detect QTLs by effectively capturing the combined contribution of multiple marker effects within a specific region. This approach enables the identification of genetic variants that may have modest effects individually but collectively contribute to the trait’s variation as well as rare variants whose effects are difficult to capture because of lack of statistical power (Oppong et al., 2022). Consequently, there is a benefit to be gained in terms of improving heritability estimates and uncovering genetic variants involved in the control of traits by fitting genome-wide analytical models that adequately capture the combined effects of rare genetic variants (Shirali et al., 2016).

We partitioned the genome of 31 + X chromosomes of the horse into 4,769 windows with the size of 90 SNPs. On average we assumed each window covered ∼0.5 Mb of the genome. To determine the optimal window size, we used the longest chromosome in the horse genome (ECA1) as a reference, which has a length of 188.3 Mb. We calculated the SNP density on this chromosome by dividing the total number of distributed SNPs by its length. The result was approximately 190 SNPs per Mb. Based on this, we selected a window size of 0.5 Mb, which corresponds to ∼90 SNPs. Then we ran a logistic linear mixed model with two variance components by considering the following mixed model:

$$Y=X\beta +Z_{w}g_{w}+Z_{r}g_{r}+\varepsilon$$

Where **Y** is the vector of DSLD case-control status as the binary phenotype, X is a design matrix of fixed effects, and β is a vector of fixed effects, $Z_{w}$ and $Z_{r}$ are the design matrices for the local window (w) and the rest of genome (r) random effects, respectively, with distributions and covariance structures of $g_{w}∼\;N\;\left(0,G_{w}\sigma_{g_{w}}^{2}\right)$ , $g_{r}∼\;N\;\left(0,G_{r}\sigma_{g_{r}}^{2}\right)$ and $\varepsilon ∼\;N\;\left(0,I\sigma_{\varepsilon }^{2}\right)$ . Here, $G_{w}$ and $G_{r}$ were the relationship matrices calculated using markers that were in the window and all SNPs out of that given window. We then selected the top 5% of windows with the highest heritability and searched for DSLD candidate genes influencing disease risk in each window through the UCSC genome browser using the EquCab3.0 reference genome. Candidate genes were then investigated for relevance to tendon homeostasis using PubMed and the search term “tendon”.

### 2.7 Signatures of selection (SOS) analysis

Evidence of signatures of positive selection across the genome of case and control groups was investigated through five complementary statistics designed to detect signatures of selection, including nucleotide diversity (Δπ), number of segregating sites by length (nSL), a statistical test based on a measure of haplotype homozygosity (H12), and integrated haplotype score (iHS). The different statistics were combined using the decorrelated composite of multiple signals method (DCMS) (Ma et al., 2015). This method combines signals of multiple tests and considers potential correlations among the different tests to increase resolution and reduce the proportion of false positives. Nucleotide diversity (π) was calculated with vcftools (Danecek et al., 2011) and the other statistics were calculated using selscan and normalized using the norm script as implemented in selscan (Szpiech & Hernandez, 2014). For each statistic within the case and the control groups, we computed the *P*-value of the DCMS statistic using fractional ranks using the stat_to_pvalue () function in the MINOTAUR R package (Verity et al., 2017) for all of the SNPs. Then, using the covNAMcd function (alpha = 0.75, nsamp = 50,000) from the rrcovNA R package to calculate an s  ×  s correlation matrix (i.e., the minimum covariance determinant estimator of multivariate location and scatter) between the included statistics (where s represents the number of statistics to estimate the DCMS values). This matrix was used as input in the DCMS function of the MINOTAUR R package to calculate genome wide DCMS values. Once the DCMS values were generated, they were fitted to a normal distribution using the robust linear model (rlm) function of the MASS R package in model = rlm (dcms ∼ 1), in which the dcms object is a vector containing the raw DCMS values. The outputs of the fitted model (i.e., Mu [mean] and SD [standard deviation]) were used as input in the pnorm R function to calculate *P*-values for the DCMS statistics: dcms_pvalues = pnorm (q = dcms, mean = Mu, sd = SD, lower.tail = FALSE). SHAPEIT2 (Loh et al., 2016) was used for haplotype phasing of autosomes, separately for case and control groups. A list of SOS regions was developed by using the EquCab3.0 genome assembly on the Ensemble genome browser. Regions of the reference genome were scanned ±50 kb upstream and downstream from the SNPs exhibiting a positive selection signature. The analysis aimed to identify genes within the candidate regions that exhibited selection signatures specifically in the cases as the target cohort, rather than in both cases and controls. Candidate genes influencing disease risk were then investigated for relevance to tendon homeostasis using PubMed and the search term “tendon”.

### 2.8 Pathway enrichment analysis

In the next step, the genes identified in the top 5% of genomic regions identified from the local genomic variance analyses using GWAS, regional heritability, and SOS analyses underwent functional analysis using genes with biological relevance to tendon homeostasis. This step aimed to reduce potential bias and increase the specificity of our analysis, by screening out unrelated genes based on prior knowledge, functional annotations, and available literature on DSLD and related biological processes. By doing this, we aimed to focus our analysis on genes that were more likely to be directly involved in the condition.

The gene lists derived from the GWAS, SOS and local window variance data were used for pathway enrichment analysis, using g:Profiler (https://biit.cs.ut.ee/gprofiler/), to identify Reactome pathways that are enriched in the experiment. The false discovery rate was set at 0.05 (Raudvere et al., 2019). Pathway enrichment analysis results were visualized and interpreted in Cytoscape using its EnrichmentMap plugin (http://www.baderlab.org/Software/EnrichmentMap) (Shannon et al., 2003).

### 2.9 Principal component analysis (PCA)

We assessed the genetic diversity within the Peruvian horse population using PCA. This analysis enabled us to investigate the variation present within the population and gain insight into its genetic structure. To accomplish this, the genotypic information was also used to compute a GRM between all individuals in case and control groups (VanRaden, 2008). By performing eigen decomposition of the GRM using the base eigen () function in R (R Core Team, 2021), the eigenvectors and eigen values were obtained and the eigenvectors were normalized. Finally, PCs were computed by multiplying eigenvectors by the square root of the associated eigenvalues (Bryant & Yarnold, 1995; Momen et al., 2022). To review the results, we plotted the projection of the individuals on the first two PCs, with colors corresponding to their group assignment.

### 2.10 Polygenic risk score prediction of DSLD risk

#### 2.10.1 Machine learning models

Four different machine learning models: weighted subspace random forest (RF), gradient boosting machine (GBM), least absolute shrinkage and selection operator (LASSO), and elastic net (EN) were used to predict DSLD polygenic risk scores. A weighted RF model was used because the weighted form of the RF model can achieve high accuracy in classifying high dimensional data (Banfield et al., 2006), such as datasets with thousands of SNPs. Because such data often contain many uninformative features for classification, random sampling often does not include informative features in selected subspaces. So, the Weighted Subspace Random Forest algorithm (wsrf) (Xu et al., 2012) and the wsrf R package was used. The gradient boosting algorithm was the second machine-learning algorithm used. It has been shown that this algorithm has a similar or higher predictive accuracy than traditional methods, in both classification and regression problems (Friedman, 2002). The boosting algorithm has been previously used in genome-wide prediction and disease susceptibility studies in animal and plant breeding (Momen et al., 2018; Montesinos- López et al., 2022). A GBM model which combines predictions from an ensemble of tree-based classifiers for outcome prediction (Chen and Guestrin, 2016) to generate the final predictions was the algorithm used as a classifier and the R package gbm (Greenwell et al., 2019) was used for implementation. Tuning of the hyperparameters was performed using a 10-fold cross validation grid search technique. Model training and optimization of tuning parameters used the caret R package (Kuhn, 2015).

The third model was the LASSO approach (Tibshhirani and VanRaden, 1996) which is used for efficient feature selection based on the assumption of linear dependency between input features and output values. In a general form, the lasso estimator uses the ℓ1 penalized least squares criterion to obtain a sparse solution to the following optimization problem:

$${\hat{\beta }}_{Lasso}=\mathrm{argmin}{\left\|\begin{array}{c} y-X\beta \end{array}\right\|}_{2}^{2}+\lambda {\left\|\begin{array}{c} \beta \end{array}\right\|}_{1}$$

Where, ${\left\|\begin{array}{c} y-X\beta \end{array}\right\|}_{2}^{2}=\sum_{i}^{n}{\left(y_{i}-x_{i}^{T}\beta \right)}^{2}$ , is the ℓ2 -norm (quadratic) loss function (i.e., residual sum of squares), $x_{i}^{T}$ is the i-th row of **X**, and the ${\left\|\begin{array}{c} \beta \end{array}\right\|}_{1}=\sum_{j=1}^{p}\left|\begin{array}{c} \beta_{j} \end{array}\right|$ is the ℓ1 -norm penalty on **β**, which induces sparsity in the solution, and λ≥ 0 is a tuning parameter. The ℓ1 penalty enables the lasso to simultaneously regularize the least squares fit and shrinks some components of ${\hat{\beta }}_{Lasso}$ to zero for some suitably chosen λ. The elastic net (EN) is an extension of the lasso that is robust to extreme correlations among the predictors (Friedman et al., 2010; Ogutu et al., 2012), for example, to overcome the instability of the lasso solution when SNPs as the predictors are in high linkage disequilibrium. In the EN model, there are two L1 and L2 penalties and the balance between them is controlled by a parameter (α).

$${\hat{\beta }}_{EN}=\mathrm{argmin}{\left\|\begin{array}{c} y-X\beta \end{array}\right\|}_{2}^{2}+\left(1-\alpha \right){\left\|\beta \right\|}_{2}^{2}+\alpha \lambda {\left\|\begin{array}{c} \beta \end{array}\right\|}_{1}$$

The glmnet function from the “glmnet” R-package was used for fitting LASSO and EN models. The cv.glmnet () function in this package was used to obtain optimum values for α and λ using a cross validation procedure.

#### 2.10.2 Bayesian regression prediction models

Four Bayesian regression models included Bayesian ridge regression (BRR), Bayes B (BB), Bayes C (BC) and Bayesian Lasso (BL) models were fitted and compared in terms of their prediction accuracy. We assumed that there is a genomic variable predictor, i.e., $G=\left\{g_{ij}\right\}$ with i=1,…,n , $j=1,\ldots ,p_{g}$ . The phenotypic vector $\left.y={\left\{y\right.}_{i}\right\}$ was defined as either $y_{i}=0$ for phenotype-negative controls or $y_{i}=1$ for DSLD cases. A probit link function as $P\left(y_{i}=1|G_{i}\right)=\Phi \left(\eta_{i}\right)$ was used to estimate the model parameters. Where, Φ is a standard normal cumulative distribution function and $\eta_{i}$ is a linear predictor that has the following form:

$$\eta_{i}=\mu +\sum_{1}^{p_{g}}g_{ij}\beta$$

Where, *µ* is an intercept or population mean, $g_{ij}$ is the genotype of the i-th individual at the j-th marker, and $\beta_{j}$ is the j-th marker effect. The probit link implemented used a latent normally distributed variable $l_{i}=\eta_{i}+\varepsilon_{i}$ and a measurement model $y_{i}=0$ if $l_{i}<\gamma$ , and 1 otherwise, where γ is a threshold parameter; $\varepsilon_{i}$ is an independent normal model residual with mean zero and with variance set equal to one. A standard Bayesian linear model was used for prediction as follows:

$$p\left(\theta_{g}|y,\omega_{g}\right)\propto p\left(y|\theta_{g}\right)\;p\left(\theta_{g}|\omega_{g}\right)$$

Here, $p\left(\theta_{g}|y,\omega_{g}\right)$ is the conditional posterior density of the genomic parameters ( $\theta_{g}=\left\{{\mu ,\sigma }_{e}^{2},\beta \right\}$ ), including the residual variance ( $\sigma_{e}^{2}$ ), which was assigned a scaled-inverse χ^2^ prior density, *µ* was assigned a flat prior density, and the marker effects ( β ) were assigned independent and identically distributed informative priors, depending on the model, and $\omega_{g}$ is the genomic hyperparameter that indexes the prior density of marker effects which for the different models is: A) BRR assumes the same genetic variance for all markers; i.e., $\beta_{i}∼N\left(0,\sigma_{\beta }^{2}\right)$ . The prior distribution for marker genetic variance is the following scaled inverted chi-squared distribution, $\sigma_{\beta }^{2}|\upsilon_{\beta }S_{\beta }∼\upsilon_{\beta }S_{\beta }\chi_{\upsilon_{\beta }}^{-2}$ , with hyper-parameters $\upsilon_{\beta }$ (degrees of freedom) and $S_{\beta }$ (scale parameter) (Meuwissen et al., 2001).

BB and BC both have an extra hyperparameter ‘π’ which is the probability of a marker’s effect to be equal to zero or null and usually is assigned a Beta prior π ∼ beta ( $p_{0}$ , $\pi_{0}$ ), with $p_{0}$ > 0 and $\pi_{0}$ ϵ [0, 1] (Pérez et al., 2010). The BB model assumes the prior for marker effects follows a normal mixture distribution given by $\beta_{\text{i}}|\pi ∼\left(1-\pi \right)\text{N}\left(0,\sigma_{\beta_{\text{i}}}^{2}\right)+\pi \text{N}(0,\sigma_{\beta_{\text{i}}}^{2}=0$ ), so that, the $\sigma_{\beta_{i}}^{2}$ denotes that each SNP has its own variance with a prior distribution $\sigma_{\beta_{i}}^{2}|\upsilon_{\beta },S_{\beta }∼\upsilon_{\beta }S_{\beta }\chi_{\upsilon_{\beta_{i}}}^{-2}$ . In BC, the prior distribution for marker effects is also given by a normal mixture distribution $\beta_{\text{i}}|\pi ∼\left(1-\pi \right)\text{N}\left(0,\sigma_{\beta }^{2}\right)+\pi \text{N}(0,\sigma_{\beta }^{2}=0$ ) but assumes the same genetic variance for all markers with a prior distribution of $\sigma_{\beta }^{2}|\upsilon_{\beta },S_{\beta }∼\upsilon_{\beta }S_{\beta }\chi_{\upsilon_{\beta }}^{-2}$ which is similar to BRR assumptions.

In Bayesian LASSO, the regression parameter $\beta_{j}$ is assumed to follow a double exponential (DE) prior distribution regression (Park & Casella, 2008). In this context, $\beta_{i}|\tau_{i},\sigma_{e}^{2}∼N\left(0,\sigma_{\beta_{i}}^{2}={\tau_{i}^{2}\sigma }_{e}^{2}\right)$ , where $\tau_{i}^{2}|\lambda^{2}∼\mathrm{Exp}\left(\lambda^{2}\right)$ , the shrinkage factor λ is further assigned with a hyper prior of a Gamma distribution Gamma ( $\lambda^{2}$ |s, r), and so it can be estimated as other model parameters. Under this approach, the marginal prior distribution for the marker effect is given by $\beta_{i}|\lambda ∼Double-\mathrm{Exp}.\left(0,\lambda \right)$ . This double-exponential distribution presents higher mass at zero, but it does not necessarily set coefficients exactly to zero. The shape (s) and rate (r) parameters of the Gamma prior was specified to s = 1.1 and $r=\frac{\left(s-1\right)}{2\times \left(1-R^{2}\right)/R^{2}\times {MS}_{x}}$ (Perez and de los Campos, 2014), where ${MS}_{x}$ represents the sum of the variances of genotype values of each SNP, and R^2^ = 0.5.

We used the BGLR package (Perez and de los Campos, 2014) to fit the Bayesian regression models. A total of 200,000 iterations, plus 20,000 of burn-in samples were considered to create posterior distributions and infer the model parameters. Global convergence was checked by visual inspection of trace plots.

#### 2.10.3 Accuracy of DSLD risk PRS prediction

To find out the optimum subset of the top SNPs, based on the GWAS results, for prediction of the DSLD genetic risk score, first we selected 0.5% (2,238), 1.0% (4,476 SNPs), 2.0% (8,952) and 3.0% (13,428 SNPs) and evaluated performance of all models. Overall, the best performance was when 1.00% of top SNPs was used for prediction.

Five-fold cross validation was used to investigate accuracy of DSLD risk prediction. We used the coefficients of determination (R^2^) on the probit and logit liability scale to assess predictive performance of our models. R^2^ on the liability scale can be obtained by transforming R^2^ on the observed scale from linear regression, using the Robertson transformation (Dempster and Lerner, 1950) as:

$$R_{l}^{2}=R_{o}^{2}\;\frac{K\left(1-K\right)}{z^{2}}$$

Where, $R_{o}^{2}$ is on the observed scale, Z is the height of a normal density curve at the point according to the population prevalence of the disease, and K is the mean proportion of cases in the sample. In probit or logit models, the above formula can be directly obtained as the proportion of variance explained by linear predictors in relation to the total variance on probit liability scale as:

$$R_{probit}^{2}=\frac{var\left({\hat{b}}_{probit}g_{i}\right)}{var\left({\hat{b}}_{probit}g_{i}\right)+var\left(e\right)}$$

where $var\left({\hat{b}}_{probit}g_{i}\right)$ is the variance due to the explanatory variable (genetic variance) and residual variance is defined as var(e) = 1. Also, on the logit scale, R^2^ can be obtained by residual variance of var(e) = $\frac{\pi^{2}}{3}=3.92$

$$R_{logit}^{2}=\frac{var\left({\hat{b}}_{logit}g_{i}\right)}{{var(\hat{b}}_{logit}g_{i})+var\left(e\right)}$$

These formulas allowed us to quantify the proportion of the total variance in the liability scale that can be attributed to the genetic factors captured by the linear predictors in the probit and logit models (Lee et al., 2012). So, by analyzing the power of our models’ R^2^ values to the total variance in the liability scale, we gain insights into the predictive power of our models and their ability to explain the underlying genetic variation.

Furthermore, to validate the predictive performance of the models, a separate validation set comprising 10 Peruvian Horses with known DSLD case (*n* = 3) and control (*n* = 7) phenotypes was utilized. Ensemble prediction was applied, incorporating all eight prediction models (BRR, Bayes B, Bayes C, BL, RF, GB, LASSO, EN). Finally, tuning of the posterior probability threshold for PRS (Polygenic Risk Score) prediction as a DSLD case was carried out using the validation set.

## 3 Results

### 3.1 Clinical findings in the study population

Pituitary pars intermedia dysfunction (PPID) was identified in two DSLD cases and two phenotype-negative controls, and equine metabolic syndrome (EMS) was identified in four DSLD cases and three controls from medical records (Table 1). No control horses were reassigned as cases.

**TABLE 1:** Clinical features of the Peruvian Horse study population.

Phenotype	DSLD cases (80 horses)	DSLD phenotype-negative controls (103 horses)
Male	7	10
Gelding	26	48
Female	46	45
Unknown	1	0
Age (years)	16.2 ± 5.3	19.1 ± 4.3
PPID	2	2
EMS	4	3

Note: DSLD, degenerative suspensory ligament desmitis; PPID, pituitary pars intermedia dysfunction; EMS, equine metabolic syndrome.

### 3.2 Heritability

**FIGURE 2:**
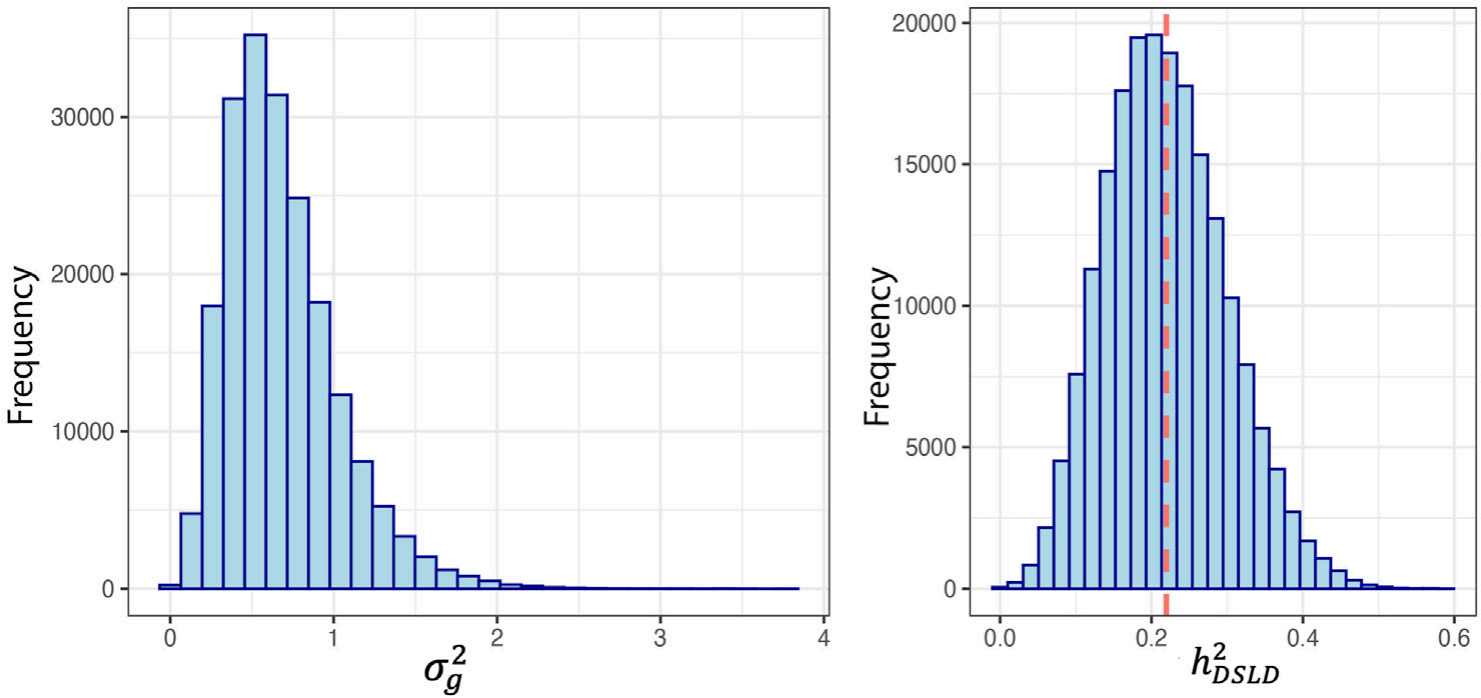
Posterior densities for genetic variance component and heritability of degenerative suspensory ligament desmitis (DSLD) in the Peruvian Horse. The red line denotes the mean (SD) of the heritability distribution. The heritability estimate was 0.22 ± 0.08 and the mean (SD) of the genetic variance was 0.68 ± 0.34.

Heritability analysis included 1,947 Peruvian Horses and was conducted using pedigree information. There were 499 (25%) horses considered inbred based on the F coefficient. The mean F coefficient was 1.72% and showed a range from 0.012% to 28.2%. The mean F in the inbred horses was 6.1%. The sample population included 688 founder horses and 1,259 non-founder horses. There were 376 horses with no progeny and 1,570 horses with at least one progeny. The posterior density of the estimated genetic variance $\sigma_{g}^{2}$ , and DSLD heritability ( $h_{DSLD}^{2}$ ) are represented in Figure 2. The posterior mean ± SD of DSLD heritability was 0.22 ± 0.08 with the highest (posterior) density interval (HPD) of lower and upper limits 0.081 and 0.419 respectively at 0.95 percent credible interval. The genetic variance component had a posterior mean and standard error of 0.683 ± 0.341, with the HPD interval’s boundary of 0.134–1.371.

### 3.3 Genome-wide association study and regional window variance

**FIGURE 3:**
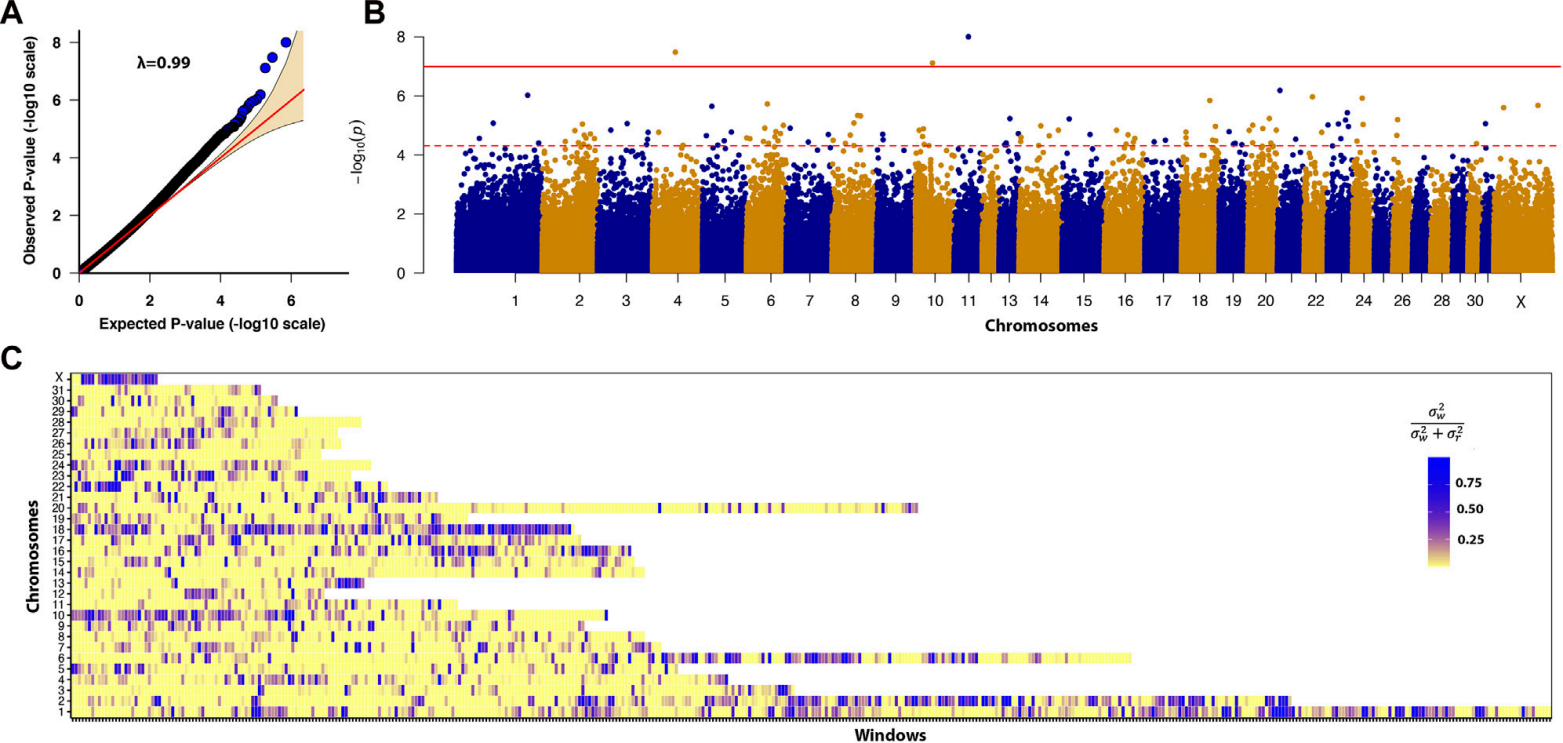
**(A)** Quantile-quantile plot comparing the expected *P*-value distribution to the observed P-value distribution. **(B)** Manhattan plot of -log_10_ (*P*-value). A linear mixed model GWAS analyzed the association between 447,630 SNPs and the DSLD disease phenotype. The solid red line denotes the Bonferroni corrected significance threshold of ≤1.1E-07. The dotted red line denotes the permutation significance threshold of ≤7.39E−05. There were 3 SNPs that passed the Bonferroni corrected *P*-value threshold and 151 SNPs that passed the permutation threshold. **(C)** Enriched heritability windows were evident in multiple regions across the genome on chromosomes 1, 2, 6, 10, 13, 16, 18, 22, and the X chromosome.

The study population consisted of 80 cases and 103 controls. There were 7 and 10 stallions, 46 and 45 mares, and 26 and 48 geldings in the case and control groups respectively. The neuter status of one male horse in the case group was unknown. In our GWAS analysis we considered two cut-off thresholds, a Bonferroni corrected *P*-value threshold at *P* < 1E-7 and a permutation-based threshold at *P* < 7.39E-5. In total, 3 and 151 SNPs passed these thresholds respectively. The three SNPs that exceeded the Bonferroni significance threshold were located on chromosomes 4, 10, and 11. Candidate loci with significant SNPs that passed the Bonferroni threshold contained the *NOG*, *AHR*, and *UBE3D* genes. We identified a total of 200 DSLD candidate genes based on the less stringent permutation threshold and after we screened the gene list. After conducting a thorough functional investigation, 17 of them were considered functionally or biologically related to DSLD (Table 2). In this analysis λ = 0.99, indicating absence of systematic biases or population structure that could lead to false-positive associations. As shown in Figure 3A, only SNPs that were significantly associated with DSLD reside outside of the normal distribution line. Multiple SNP associations were identified across the genome (Figure 3B).

**TABLE 2:** Degenerative suspensory ligament desmitis candidate genes in case and control Peruvian Horses identified by genome-wide association study, signatures of selection analysis, and enriched local heritability based on the top 5% of windows consisting of 90 SNPs (∼0.5 Mb).

Chr.	Gene	Start	End	Association	Function
1	*GOT1*	30375631	30405684	WIN	Amino acid metabolism
1	*ADPGK*	121816143	121845020	WIN	Glucose metabolism
1	*HNRNPC*	159648632	159698966	WIN	mRNA processing
1	*NFATC4*	164052916	164064654	WIN	Transcription
1	*ARHGAP5*	171226123	171340624	GWAS, WIN	RhoA signaling
2	*IL2*	105981808	105986539	GWAS	Cytokine signaling
3	*CXCL1*	63470006	63471632	GWAS	Chemokine signaling
4	*GLI3*	13037249	13219199	GWAS	Mechanotransduction
4	*DLX5*	40253957	40258390	WIN	Bone development
4	AHR*	49857545	49905361	GWAS	Transcription
4	*BMPER*	64067759	64300454	GWAS	BMP signaling
5	*FMOD*	98095	108183	WIN	Extracellular matrix assembly
5	*PRELP*	216694	229790	WIN	Extracellular matrix protein
5	*PRRX1*	6933867	7004826	WIN	Transcription co-activator
5	*PLA2G4A*	20629061	20778606	GWAS	Inflammation/fibrosis
5	*DOCK7*	94635353	94839474	WIN	Neuronal homeostasis
5	*ANGPTL3*	94712964	94721879	WIN	Angiogenesis
6	*MYL1*	702203	713479	GWAS	Muscle motor protein
6	*TNS1*	7382346	7475906	GWAS	Mechanotransduction
6	*CXCR2*	7619797	7635887	GWAS	Chemokine signaling
6	*HOXC11*	71740286	71743755	WIN	Morphogenesis
6	*HOXC10*	71752515	71756888	WIN	Morphogenesis
6	*CDK2*	74578263	74584110	GWAS, WIN	Cell cycle regulator
6	*GLI1*	75986882	75997378	WIN	Sonic hedgehog signal transduction
6	*DDIT3*	76025871	76030311	WIN	Transcription factor
9	*EYA1*	14929400	15250649	GWAS, WIN	Transcriptional activator of tendongenesis
9	*FBXO32*	68005907	68040654	WIN	Ubitiquination
10	*APOE*	15712329	15715203	WIN	Fat metabolism
10	*RELB*	15824912	15849495	WIN	DNA and protein kinase binding
10	*FOSB*	16184656	16189918	WIN	Transcription
10	*C5AR1*	17637680	17648858	GWAS	Complement signaling
10	*BAX*	19185480	19189232	WIN	Regulation of apoptosis
10	UBE3D*	37554767	37708415	GWAS	Protein processing
11	NOG*	31351205	31353200	GWAS	TGF-beta signaling
12	*FAM111B*	22719710	22729624	WIN	Serine protease
13	*MMP25*	40938718	40949639	WIN	Extracellular matrix remodeling
13	*MAPK8IP3*	42229513	42283271	WIN	Protein kinase activity in the JNK pathway
14	*B4GALT7*	3762834	3769419	GWAS	Extracellular matrix homeostasis
18	*GLI2*	9941781	10145734	GWAS	Mechanotransduction
18	*ITGA4*	59353502	59429448	WIN	Cell surface adhesion and signaling
18	*ZNF804A*	61922561	62203313	WIN	Zinc finger binding protein
18	*MSTN*	66605149	66610122	WIN	TGF-beta signaling
18	*CASP8*	76419122	76440775	WIN	Apoptosis signaling
18	*BMPR2*	77363946	77575481	GWAS	BMP signaling
18	*IDH1*	82212082	82224373	WIN	Regulates cytoplasmic NADPH production
20	*DDR1*	30773386	30790979	WIN	Regulation of cell growth, differentiation, and metabolism
20	*TNXB*	32581324	32636789	GWAS, WIN	Extracellular matrix homeostasis
20	*FKBPL*	32652174	32653957	WIN	Regulation of the cell cycle
20	*MEP1A*	46257218	46290867	GWAS	Collagen type I assembly
23	*JAK2*	25863481	25998651	WIN	Cytokine and growth factor signaling
24	*TGFB3*	21412497	21434025	SOS	Regulation of SMAD transcription
24	*SYNE2*	10579333	10875981	WIN	Cell structural protein
24	*SIX1*	7880934	7885609	WIN	Limb development
X	*SMARCA1*	106902542	106969223	WIN	ATPase regulation of chromatin remodeling
X	*MIR363*	110758364	110758439	WIN	Non-coding RNA
X	*HPRT1*	110954955	110988635	WIN	Purine metabolism

Note: Candidate genes were identified through analysis of significant SNPs with ±50 kb flanking regions using the EquCab3.0 reference genome. Chr, chromosome. *Significance of association met the Bonferroni threshold. GWAS, genome-wide association study; SOS, signatures of selection; WIN, window analysis of enriched local heritability.

Multiple regions of enriched local heritability were also identified across the genome, with strong enrichment signals on chromosomes 1, 2, 6, 10, 13, 16, 18, 22, and the X chromosome (Figure 3C). In this analysis, we selected the top 5% of windows (238 windows) with the highest genetic variance and searched for the DSLD related genes in each window through the UCSC genome browser. In total 953 DSLD candidate genes were identified of which 39 genes were relevant to tendon homeostasis (Table 2).

### 3.4 Signatures of selection and principal component analysis

As a prerequisite for selection signature analysis, we performed a PCA analysis (Supplementary File S1). The results showed that the Peruvian Horses clustered together and distributed along these two vectors based on their genomic similarity with a small difference between DSLD case and control groups of horses (Supplementary Figure S1A). Our PCA analysis showed that the first principal component captured 15.9% and the second explained 5.8% of total variance (Supplementary Figure S1B).

**FIGURE 4:**
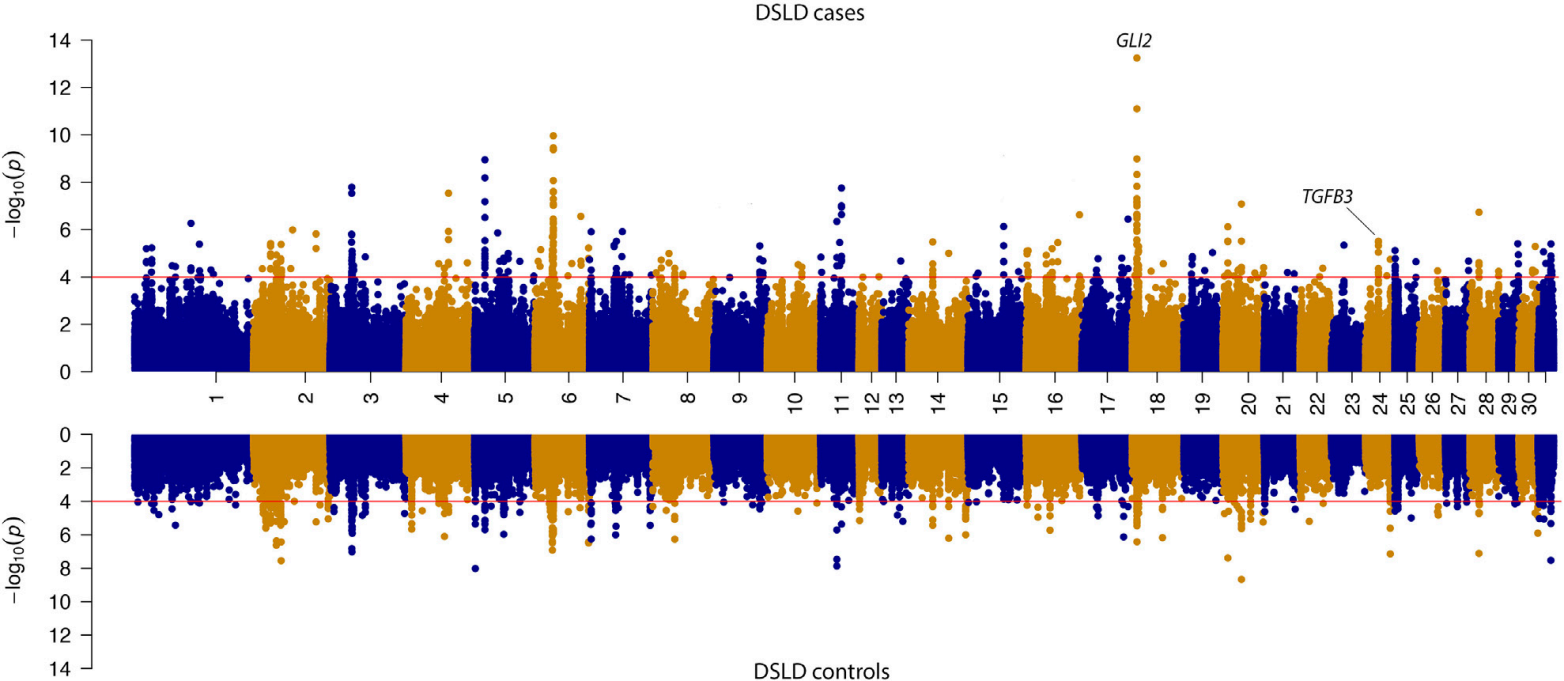
Mirrored Manhattan plot for signatures of selection results in the case and control sub-populations. -log10 *P*-value of the decorrelated composite of multiple signals (DCMS) values are shown in the upper panel for DSLD cases and in the lower panel for the control group. The red line in the figure indicates the significance cutoff of (*P*-value = 0.0001). In the DSLD case group, candidate genes included the candidate tendinopathy gene *TGFB3* that was not shared with the DSLD phenotype-negative control group. Additionally, the candidate tendinopathy gene *GLI2* exhibited a large selection signature in DSLD cases relative to DSLD controls.

The SOS analysis in case-control groups showed that there were 115 genes in candidate loci exhibiting a selection signature in DSLD cases and 123 genes in control populations. Of these genes, 49 were shared between case and control groups. In the DSLD case group, candidate genes included the candidate tendinopathy gene *TGFB3* that was not shared with the DSLD phenotype-negative control group (Figure 4 and Table 2).

### 3.5 Pathway enrichment analysis

**FIGURE 5:**
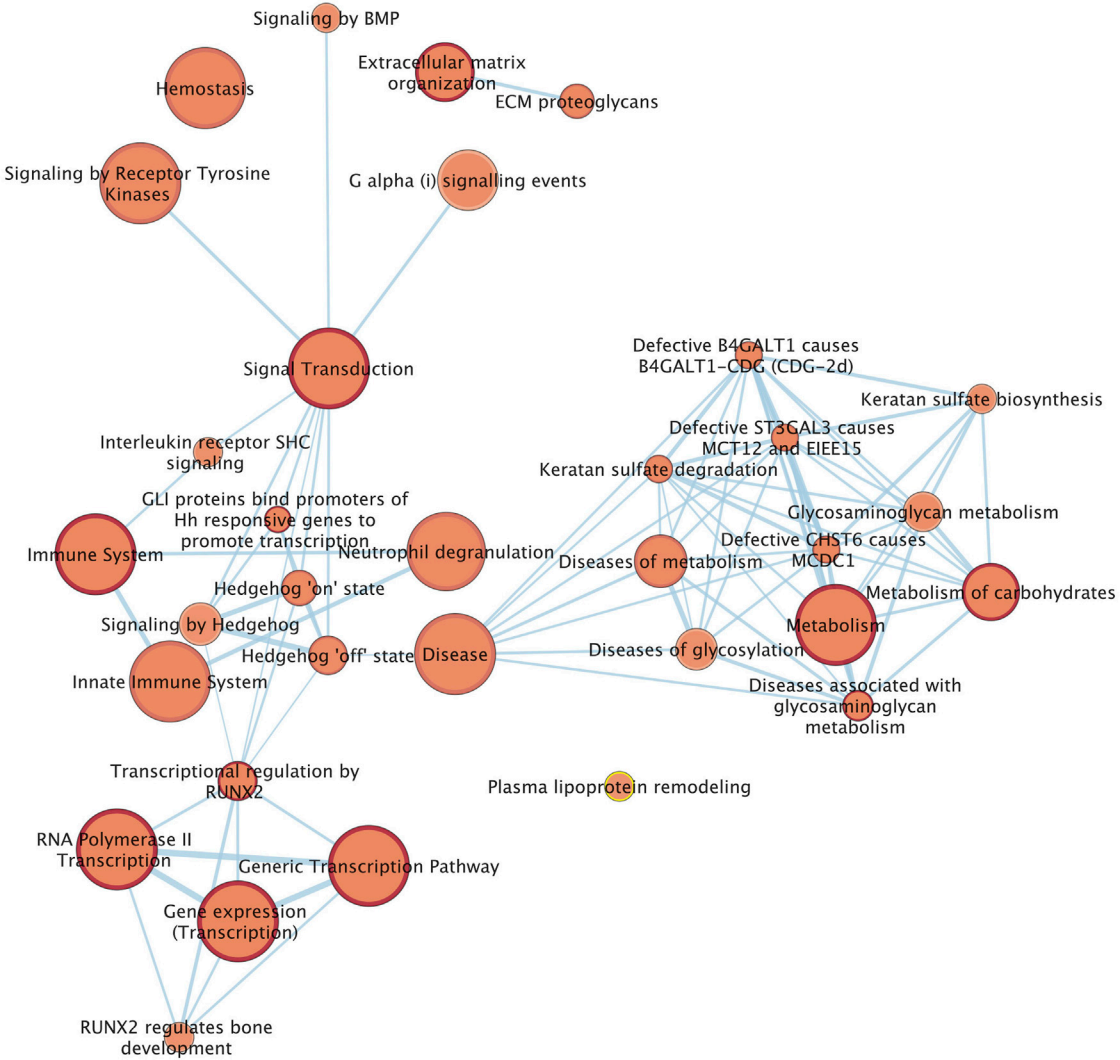
Network of the Reactome pathways. Each node (circle) represents a gene set characterized by a particular reactome pathway. Node fill indicates the enrichment score (FDR q-value). The thickness of blue lines (edges) indicates the number of shared genes (overlap) between two connected nodes. Nodes with high overlap are clustered together, forming groups characterized by similar terms and pathways.

We combined 60 candidate genes obtained from the three analyses (20 genes from GWAS, 39 genes from window based local variance analysis, and one gene from SOS analysis), for Reactome pathway enrichment analysis. Finally, 33 pathways from the Reactome data base which were most related to tendon homeostasis, were identified (Figure 5). SNP associations with DSLD showed enrichment for pathways including proteoglycan metabolism, extracellular matrix homeostasis, and signal transduction pathways that included the hedgehog signaling pathway (Figure 5).

### 3.6 Polygenic risk score prediction of DSLD risk

Predictive performance of both classification machine learning and Bayesian regression models using a five fold cross validation is represented in Table 3. Machine learning performance was assessed using the top 1% of GWAS SNPs (4,476 SNPs). We considered sex as the only covariate in the predictive model. A chi-squared test with Yates’ continuity correction indicated a weakly significant association between sex and disease status in our sample population ( χ -squared = 4.55, df = 1, *p* = 0.0329). The coefficients of determination (R^2^) were estimated for the predictive performance of four machine learning classifiers and four Bayesian models on both the probit liability and logit liability scale. On the probit liability scale, the Bayesian Ridge Regression (BRR) model achieved the highest mean R^2^ of 0.702, followed closely by the Bayes B and Bayes C models with mean R^2^ values of 0.699. These models demonstrated relatively strong predictive power in explaining the variation in the data. On the other hand, the Gradient Boosting (GB) model had the lowest mean R^2^ value of 0.506, indicating relatively weaker predictive performance compared to the other models.

**TABLE 3:** The estimated coefficients of determination ( $R^{2}$ ) for each model on the probit liability scale and the logit liability scale for polygenic risk score prediction of risk of degenerative suspensory ligament desmitis in the Peruvian Horse.

	Probit liability scale $R^{2}$		Logit liability scale $R^{2}$	
Model	Mean	SD	Mean	SD
BRR	0.702	0.0176	0.718	0.0164
Bayes B	0.699	0.0189	0.717	0.0169
Bayes C	0.699	0.0183	0.716	0.0176
BL	0.689	0.0198	0.704	0.0213
RF	0.632	0.0381	0.606	0.0363
GB	0.506	0.0416	0.467	0.0458
LASSO	0.562	0.0173	0.525	0.0193
EN	0.682	0.0184	0.679	0.0242

Note: The Bayesian models included Bayesian Ridge Regression (BRR), Bayes B, Bayes C, and Bayesian Least Absolute Shrinkage and Selector Operator (BL). The machine learning classifiers included Random Forest (RF), Gradient Boosting (GB), Least Absolute Shrinkage and Selector Operator (LASSO), and Elastic Net (EN). The results are presented as mean and standard deviation (SD) based on the results of five-fold cross-validation.

Similar patterns were observed on the logit liability scale, where the BRR model exhibited the highest mean R^2^ of 0.718, followed by Bayes B and Bayes C with mean R^2^ values of 0.717 and 0.716, respectively. The GB model again showed the lowest mean R^2^ of 0.467, suggesting comparatively lower predictive accuracy. Among the machine learning classifiers, Random Forest (RF) had a mean R^2^ of 0.632, while LASSO and Elastic Net (EN) achieved mean R^2^ values of 0.562 and 0.682, respectively on the probit liability scale.

When PRS prediction of a separate validation set of Peruvian Horses was performed using ensemble risk prediction, four DSLD control horses were incorrectly predicted using a posterior probability threshold of 0.5 for classification as a case (Supplementary Table S1). Tuning of the threshold (Supplementary Figure S4) identified an optimal threshold of 0.55. With this adjusted threshold, correct classification was achieved for 9 out of 10 horses (Supplementary Table S1). One DSLD control horse was still predicted to have the genetic risk of a case.

## 4 Discussion

DSLD is a debilitating condition characterized by systemic deposition of proteoglycan in connective tissues that may yield insight into human TLI associated with similar matrix disturbances. We undertook a within-breed GWAS of DSLD in the Peruvian Horse using a linear mixed model to discover candidate loci and genes that influence risk of the disease. Our analysis showed that the disease has moderate heritability of 0.22 in this breed. Specific environmental risk factors for DSLD are poorly understood. Our results also show that DSLD has a polygenic architecture with risk loci spread across the autosomal genome. Novel TLI genes and pathways were highlighted from this research. PPID and EMS were occasionally identified with similar frequency in the horses in both the case and phenotype-negative control group.

Our GWAS analysis identified 151 DSLD-associated SNPs, suggesting DSLD is a complex polygenic disease. Environmental risk factors account for the remaining risk. Candidate loci with significant SNPs that passed the Bonferroni threshold contained the *AHR*, *NOG* and *UBE3D* genes. The *AHR* gene regulates transcription *via* the aryl hydrocarbon receptor. A role in tendon biology has not been defined, but it is possible that this gene may have a role in extracellular matrix degradation during aging (Salminen, 2022). *NOG* is a 222 amino acid secreted protein known for binding and inactivating *BMP4* and other proteins in the transforming growth factor beta (TGF) superfamily. *NOG* is known to play an important role in tendon development and homeostasis (Schweitzer et al., 2001), including development of heterotopic ossification with tendon aging (Dai et al., 2020). *BMP2*, another member of the *TGF* superfamily, has been previously identified within cellular foci of fibroblasts in DSLD-affected SL (Young et al., 2018). *BMPER* and *BMP2R* were also identified as candidate genes in this analysis. DSLD is associated with an atypical accumulation of proteoglycans, such as aggrecan, within diseased SL tissue (Plaas et al., 2011). It is conceivable that *NOG* may influence aggrecan homeostasis in SL tissue matrix through BMP-SMAD1/5 signaling (Wang et al., 2012). *UBE3D* is a ubiquitin-conjugating enzyme that plays an important role the ubiquitin proteosome system, regulating protein degradation. It is possible that functional variation in this protein may contribute to the pathogenesis of DSLD through protein degradation (Huang et al., 2015).

A much larger number of SNPs passed the permutation threshold used in this study. Bonferroni correction is widely considered too conservative and may propagate Type II error (false negatives) (Nakagawa, 2004). Because groups of SNPs are inherited together in a haplotype block because of linkage disequilibrium, association testing of individual SNPs is not independent. In this larger set of SNP associations, additional DSLD risk SNPs were identified in genes that could influence tendon homeostasis. Increased *CXCL1* expression has been found in chronic tendinopathy (Kendal et al., 2020). *GLI2* and *GLI3* were also identified as candidate DSLD genes. *GLI3* has been linked to mechanotransduction responses during tendon healing (Freedman et al., 2022). In humans, increased expression of *MYL1* has been identified in traumatic rotator cuff tears in female patients, whereas *MYL2* is highly expressed in degenerative tears in male patients (Rai et al., 2022). A mutation in *B4GALT7* has been associated with dwarfism and development of tendon laxity in Friesian horses. B4GALT7 is one of the enzymes that synthesizes the tetrasaccharide linker between protein and glycosaminoglycan moieties of proteoglycans in extracellular matrix (Leegwater et al., 2016). A mutation in *TNXB* has also been associated with connective tissue laxity that is part of an Ehler-Danlos Syndrome-like phenotype (Brisset et al., 2020). Tenascin-X is a matrix glycoprotein that is thought to have an important role in collagen fibrillogenesis (Brisset et al., 2020). Collagen assembly is also regulated by *MEP1A* (Broder et al., 2013), another DSLD-candidate gene identified in this research.

Through our SOS analysis, we found 66 candidate genes that exhibited a selection signature in DSLD cases that was not present in the control group, further supporting the hypothesis that DSLD has a polygenic architecture. Candidate genes include 25 non-coding RNA sequences, suggesting that regulatory SNPs may play an important role in the genetic contribution to DSLD (Giral et al., 2018). We also found *GLI2* exhibited a large selection signature in DSLD cases relative to phenotype-negative control horses. A candidate genomic region from SNP GWAS that also contains a positive selection signature is more likely to contain the causal genetic variant, particularly for diseases with a simple mode of inheritance, but not for complex traits (Kemper et al., 2014). Development of tendinopathy likely represents a failure to repair or remodel extracellular matrix after repetitive micro-injury. In this regard, poor healing has been associated with loss of TGFB receptors from diseased matrix (Fenwick et al., 2001) and downregulation of *TGFB3* is found with aging, particularly in tendons exposed to mechanical overload (Kinitz et al., 2021). The Indian hedgehog signaling pathway, which includes the transcription factors *GLI1*/*GLI2/GLI3*, is known to modulate matrix responses to load and healing in tendon injury, particularly at bone attachment sites (Liu et al., 2022).

Pathway analysis of candidate genes identified by GWAS, local variance analysis and SOS identified enrichment of pathways associated with glycosaminoglycan metabolism and extracellular matrix homeostasis. Additionally, signal transduction, particularly the hedgehog signaling pathway also showed enrichment. Glycosaminoglycans have a key role in extracellular matrix composition of tendons and disturbed metabolism of the extracellular matrix of tendon; accumulation of aggrecan in SL tissue and disturbance to decorin glycosylation are key features of DSLD (Kim et al., 2010; Plaas et al., 2011; Haythorn et al., 2020). Mechanotransduction has a key role in tendon and ligament homeostasis and genes that have regulatory effects on mechanotransduction were a key finding in a previous categorical GWAS of DSLD in multiple breeds of horse (Momen et al., 2022).

The Peruvian Horse is a breed with a small effective population (Momen et al., 2022), enabling detection of significant associations and accurate PRS predictions with a relatively small sample size. In the cross-validation experiment, the models we studied demonstrated moderate predictive performance on both the probit and logit liability scales. It is important to consider both the mean R^2^ values and the corresponding SD, representing the variability of R^2^ values across the cross-validation folds. The low SD values indicate more stable and consistent predictive performance. We identified the BRR model as the best performing single model with a clinically relevant predictive accuracy in the reference population with an R^2^ that exceeds 0.7 using the top GWAS SNPs and sex as the only covariate in the predictive model. When we used ensemble prediction in a validation set of 10 independent Peruvian Horses, all horses were predicted accurately except one after tuning of the posterior probability threshold.

These results fit with an earlier observation that PCA analysis using top DSLD GWAS SNPs reflecting breed categorical risk causes the population within the Peruvian Horse breed to form two distinct clusters in contrast to a single breed cluster when all SNPs are considered in the analysis (Momen et al., 2022). Because DSLD is an acquired disease that often develops after horses have reached breeding age and been used for breeding, accurate PRS prediction of DSLD risk is an important advance clinically, as it enables screening of horses for selection for breeding at a young age.

There were several limitations to this work. The sample size in our study population was relatively small at 183 horses. The age was not available for all the cases. In the future, increasing the sample size may help detect additional associations, improve the accuracy of PRS prediction of disease risk, and enable SNP estimation of DSLD heritability. Consideration of athletic activity in prediction models may also be useful in the future. Further validation of PRS prediction is needed by predicting a larger independent test set of horses and evaluating prediction accuracy. It would also be important to follow predicted horses over time to confirm whether young horses predicted as cases develop DSLD later in life. Combining both genotype and pedigree data to estimate heritability could also be considered. In our regional window variance analysis, the choice of the threshold for selecting the top windows with the highest heritability was a subjective decision. A higher or lower threshold than 5% may have yielded different results. More work is also needed to further investigate the key pathways involved in the pathogenesis. RNA-Seq analysis of tendon tissue from DSLD case and control horses will likely help confirm key candidate genes and pathways. Further investigation of candidate genetic variants using whole genome sequencing is also warranted. By including all genes related to DSLD from GWAS, SOS and WIN, and shortening the gene list by selecting biologically compelling genes, we aimed to capture a set of genes that are biologically relevant to the phenotype of interest and increase the power of our pathway analysis to detect meaningful associations. Additionally, we assumed this approach would help reduce the impact of false negatives in our analysis compared with inclusion of all associated genes from our discovery analyses.

In conclusion, our within-breed GWAS of DSLD in the Peruvian Horse has further confirmed moderate heritability and a polygenic architecture underlies the trait and identified multiple DSLD SNP associations. Pathways enriched with DSLD risk variants include pathways that influence glycosaminoglycan metabolism, extracellular matrix homeostasis, signal transduction, interleukin signaling, and apoptosis. PRS prediction using an ensemble prediction pipeline shows clinical promise as a genetic risk test for DSLD.

## Data Availability

The coded sex phenotype and genotypic data used for this project are available via the Dryad Digital Repository: https://doi.org/10.5061/dryad.cnp5hqc9p. The degenerative suspensory ligament desmitis (DSLD) case or control phenotypes of the Peruvian Horses in the genome-wide association study SNP set are retained at UW-Madison as proprietary data.
