# Supplementary material for: Genetic architecture and polygenic risk score prediction of degenerative suspensory ligament desmitis (DSLD) in the Peruvian Horse
Source: Front Genet. 2023 Aug 14;14:1201628. doi: 10.3389/fgene.2023.1201628 (PMC10460910; doi:10.3389/fgene.2023.1201628)
Supplement: Supplementary file 1 [file DataSheet1.docx]

Supplementary Material

Genetic architecture and polygenic risk score prediction of degenerative suspensory ligament desmitis (DSLD) in the Peruvian Horse

Mehdi Momen, Kiley Brauer, Margaret Patterson, Susannah J. Sample, Emily E. Binversie, Brian W. Davis, E. Gus Cothran, Guilherme J.M. Rosa, Sabrina H. Brounts, Peter Muir^*^

***Correspondence:** Corresponding Author: peter.muir@wisc.edu

## Supplementary Figures

**
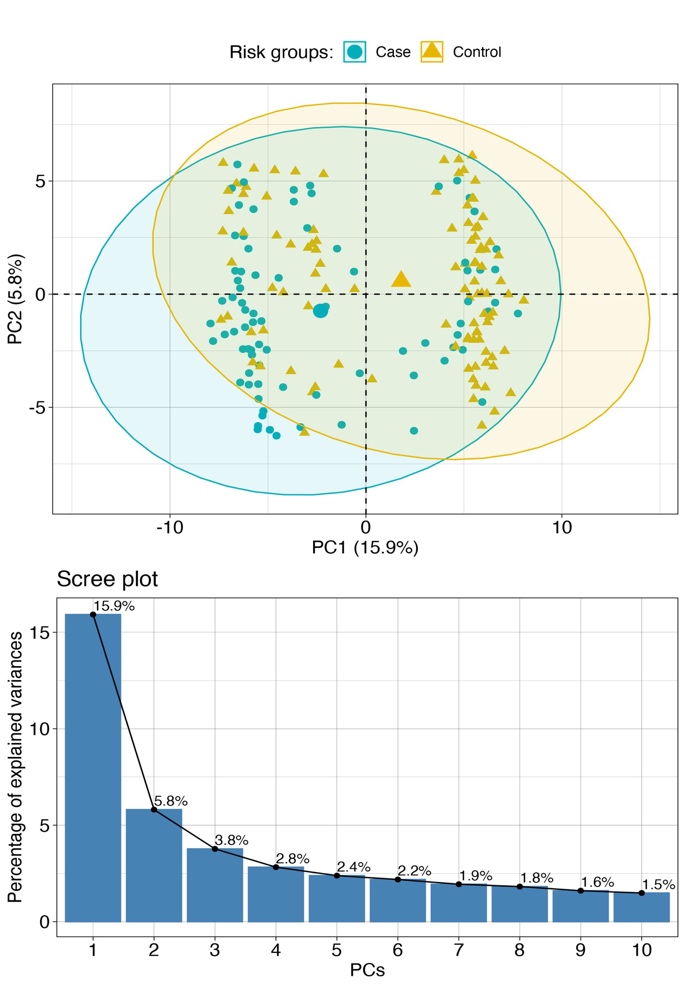
**

**B**

**A**

**Supplementary Figure 1**. Graphical representation (**A**) multi-dimensional scaling plots of the genetic distance between the degenerative suspensory ligament desmitis case and control Peruvian Horse groups. Each point corresponds to one animal and indicates the distance between animals represented by the first two principal components (PC1 and PC2), based on the genomic kinship matrices. (**B**) Scree plot of the percentage of explained variance by the first 10 PCs extracted from genomic kinship matrices

**Supplementary Figure 2**. Performance of machine learning (ML) and Bayesian (BM) prediction models over a range of top SNP percentages (0.005, 0.01, 0.02, and 0.03). The selected percentage of top SNPs that yielded higher predictive accuracy was used for the final analysis.

**Supplementary Figure 3**. (A) Quantile-quantile plot comparing the expected P-value distribution to the observed P-value distribution. The observed P-values deviate from normality Quantile-quantile plot comparing the expected P-value distribution to the observed P-value distribution. The observed P-values deviate from normality. (B) Manhattan plot of -log10 (P-value). The solid red line denotes the Bonferroni corrected significance threshold. The dotted red line denotes the permutation significance threshold. There were 3 SNPs that passed the Bonferroni corrected P-value threshold and 151 SNPs that passed the permutation threshold.


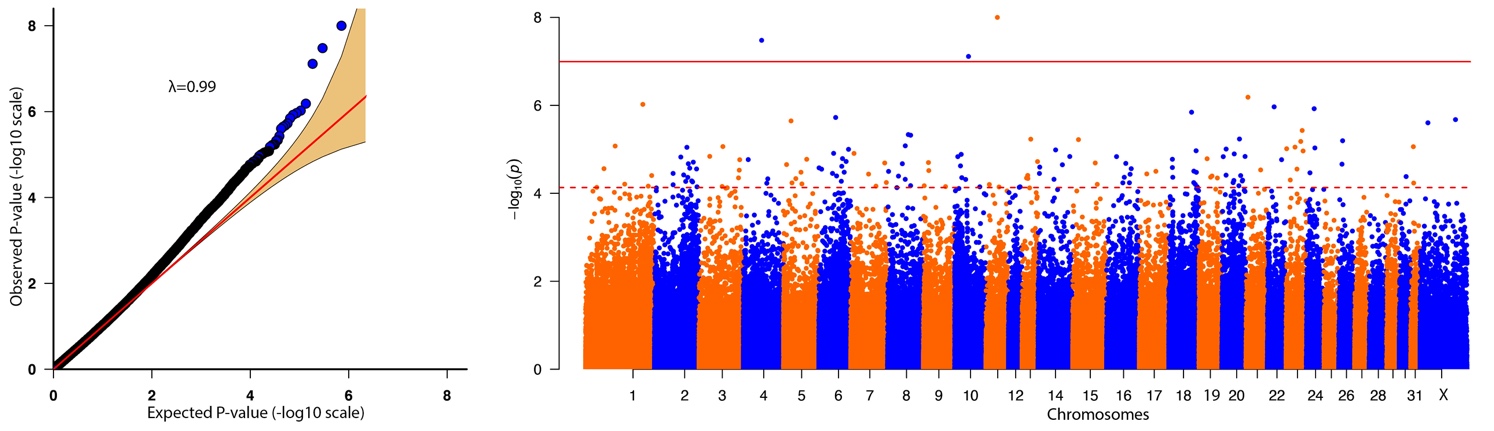


**Supplementary Figure 4**. For the independent validation test set of DSLD case and control Peruvian Horses, tuning of the posterior probability threshold ($\alpha$) was performed using three metrics. 1) p* = arg min |TPR(p)+FPR(p)-1|. 2) Accuracy = (Number of Correct Predictions) / (Total Number of Predictions). 3) G-Mean = sqrt(Sensitivity * Specificity). Based on this validation test set, this analysis yielded an optimal classification threshold of 0.55.


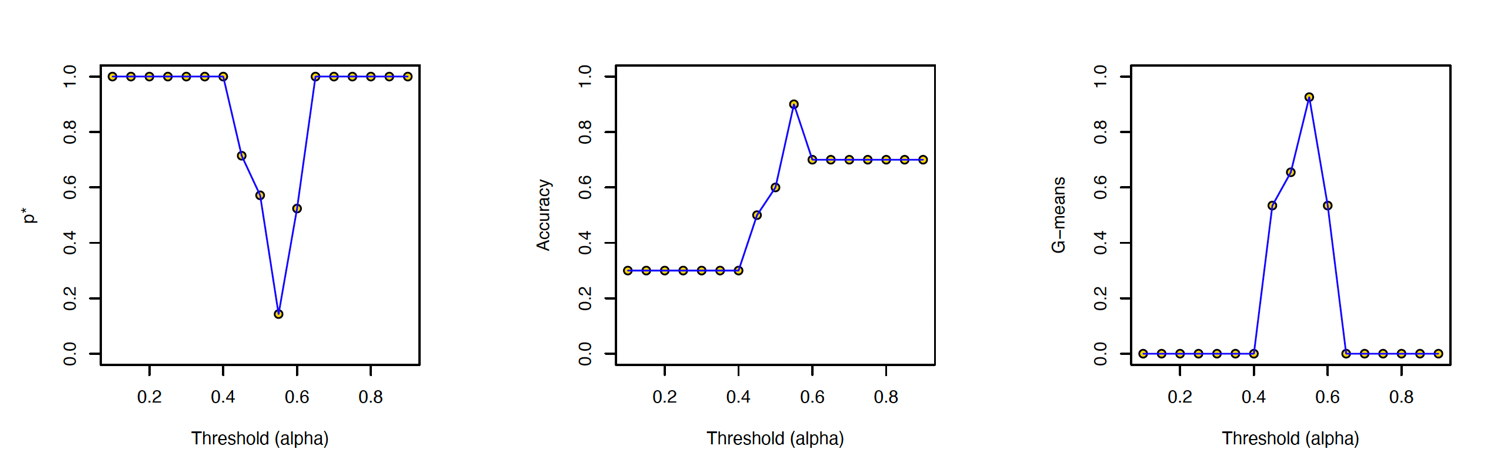


## Supplementary Tables

**Table S1**. Predicted degenerative suspensory ligament desmitis (DSLD) phenotypes from polygenic risk score (PRS) prediction using Bayesian Ridge Regression for a validation set of 10 Peruvian Horses.

| Horse | Disease status | Posterior probability being a case | Prediction  (Cut-off=0.5) | Prediction  (Cut-off=0.55) |
| --- | --- | --- | --- | --- |
| 1 | Control | 0.450 | True | True |
| 2 | Case | 0.640 | True | True |
| 3 | Control | 0.540 | False | True |
| 4 | Control | 0.440 | True | True |
| 5 | Control | 0.537 | False | True |
| 6 | Case | 0.590 | True | True |
| 7 | Case | 0.573 | True | True |
| 8 | Control | 0.637 | False | False |
| 9 | Control | 0.477 | True | True |
| 10 | Control | 0.538 | False | True |

# Note. The prediction model used was ensemble prediction using four Bayesian and four machine learning statistical models (Bayesian Ridge Regression (BRR), Bayes B, Bayes C, Bayesian Least Absolute Shrinkage and Selector Operator (BL), Random Forest (RF), Gradient Boosting (GB), Least Absolute Shrinkage and Selector Operator (LASSO), and. Elastic Net (EN)).
